# The prognosis of patients with locally advanced cervical cancer undergoing surgical versus non-surgical treatment: a retrospective cohort study based on SEER database and a single-center data

**DOI:** 10.1097/JS9.0000000000002098

**Published:** 2024-10-01

**Authors:** Xinyan Gao, Yan Kong, Ying Ning, Tian Tian, Xiaojing Gai, Ke Lei, Zhumei Cui

**Affiliations:** a School of Clinical Medicine, Qingdao University, Qingdao, China; b Department of Gynecology, The Affiliated Hospital of Qingdao University, Qingdao, China; c Qingdao Women and Children’s Hospital, Qingdao, China; d Center of Tumor Immunology and Cytotherapy, Medical Research Center, The Affiliated Hospital of Qingdao University, Qingdao, China

**Keywords:** chemoradiotherapy, locally advanced cervical cancer, prognosis, surgery

## Abstract

The aim of this study was to investigate the impact of surgical treatment on the survival prognosis of patients with locally advanced cervical cancer (LACC) and to identify factors that may influence the efficacy of surgery. Data from the SEER database (2000–2020) and a hospital (2013–2023) were collected for this investigation. Utilizing multivariable Cox regression analysis, Kaplan–Meier survival analysis, and log-rank tests, we assessed the effects of surgical intervention on overall survival (OS) and disease-specific survival (DSS) in LACC patients. Our results revealed that in the SEER database, the surgical group exhibited significantly better OS and DSS compared to the non-surgical group. Particularly noteworthy was the significantly higher survival rate in the surgical group for patients with tumor diameters less than 6 cm. Furthermore, both OS and DSS were improved in the surgical group regardless of whether the cancer was squamous cell carcinoma or adenocarcinoma. Additionally, patients who underwent surgery combined with radiotherapy had notably better OS and DSS compared to those who received chemoradiotherapy alone. Similarly, our hospital data showed that the surgical group demonstrated significantly better OS than the non-surgical group, especially for patients with tumors smaller than 6 cm in diameter. These findings suggest that surgery combined with radiotherapy may offer more favorable outcomes than chemoradiotherapy alone, particularly for LACC patients with smaller tumors.

## Introduction

Highlights

- This study comprehensively evaluated the prognostic efficacy of surgical treatment in patients with locally advanced cervical cancer.
- Surgery improved OS and DSS in LACC, especially for tumors <6 cm.
- Age, histology, tumor grade, and TNM stage were not limiting factors in the choice of surgical treatment.

Cervical cancer is a prevalent form of malignancy among women worldwide and ranks as the fourth leading cause of cancer-related deaths among women, posing a significant threat to women’s health globally^1^. According to the 2018 International Federation of Gynecology and Obstetrics (FIGO) staging system, narrowly defined LACC falls primarily under stage IB3 and IIA2. While the National Comprehensive Cancer Network (NCCN) guidelines recommend concurrent chemoradiotherapy as the primary treatment for LACC patients, with radical surgery as a secondary option, variations in radiotherapy quality between hospitals can lead to significant differences in patient outcomes. Previous studies by Ryu in 2007^2^ and Bradbury in 2015^3^, comparing various treatment modalities for LACC, found that patients who underwent initial surgery had the highest survival rates. Surgical resection of lesions distant from the radiation field may enhance long-term OS and DSS; however, this approach also increases the risk of postoperative complications and financial burden^4,5^. Therefore, there is ongoing debate regarding whether LACC patients should undergo surgical intervention.

## Methods

Data on patients diagnosed with primary LACC from 2000 to 2020 were collected from the SEER database, and additional data were gathered from a hospital for the period 2013–2023. A total of 2759 SEER database patients and 181 hospital patients met the inclusion criteria. Patients who did not receive radiotherapy or surgery or were not staged as IB3 or IIA2 were excluded from the analysis.

Surgical information was classified into surgical and non-surgical groups based on SEER program codes and the 2023 staging manual. The surgical group included patients who underwent various hysterectomy procedures, while the non-surgical group consisted of patients who received no surgical treatment or only local cervical procedures.

The primary endpoints of this study were OS and DSS. OS was defined as the time from diagnosis to death from any cause, while DSS was defined as the time from diagnosis to death, specifically due to cervical cancer.

Continuous variables were described using means±standard deviations and compared between groups using the Mann–Whitney *U* test. Categorical variables were described using frequencies and percentages and compared using the Chi-square test or Fisher’s exact test as appropriate.

Kaplan–Meier survival curves were used to illustrate changes in OS and DSS under different treatment modalities, with the log-rank test employed to compare differences between the curves. To identify independent prognostic factors influencing OS and DSS, multivariable Cox regression analysis was performed, with hazard ratios (HRs) and 95% confidence intervals (CIs) used to assess the impact of variables on survival outcomes.

## Results

Analysis of the SEER database revealed that the surgical group demonstrated significantly better 5-year OS and DSS compared to the non-surgical group. Specifically, for patients with tumors smaller than 6 cm, the surgical group exhibited a significantly higher 5-year OS (81.2% vs. 71.4%, *P*<0.0001) and 5-year DSS (87% vs. 79.3%, *P*<0.0001) than the non-surgical group. Furthermore, regardless of whether the cancer was squamous cell carcinoma or adenocarcinoma, the surgical group showed superior OS and DSS outcomes compared to the non-surgical group.

Patients who received surgery combined with radiotherapy had significantly better 5-year OS and DSS than those who received chemoradiotherapy alone.

**Figure 1:**
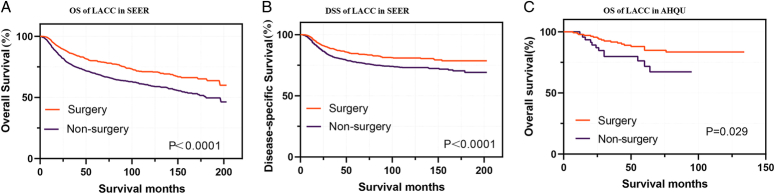
Survival curves for patients with LACC: surgical treatment vs. non-surgical treatment. (A) Overall survival (OS) of patients with LACC in the SEER database; (B) disease-specific survival (DSS) of patients with LACC in the SEER database; (C) OS of patients with LACC in the affiliated hospital of Qingdao University (AHQU) database.

Similarly, in the hospital dataset, the surgical group exhibited significantly better OS compared to the non-surgical group, particularly for patients with tumors less than 6 cm in diameter (Fig. 1).

**Figure 2:**
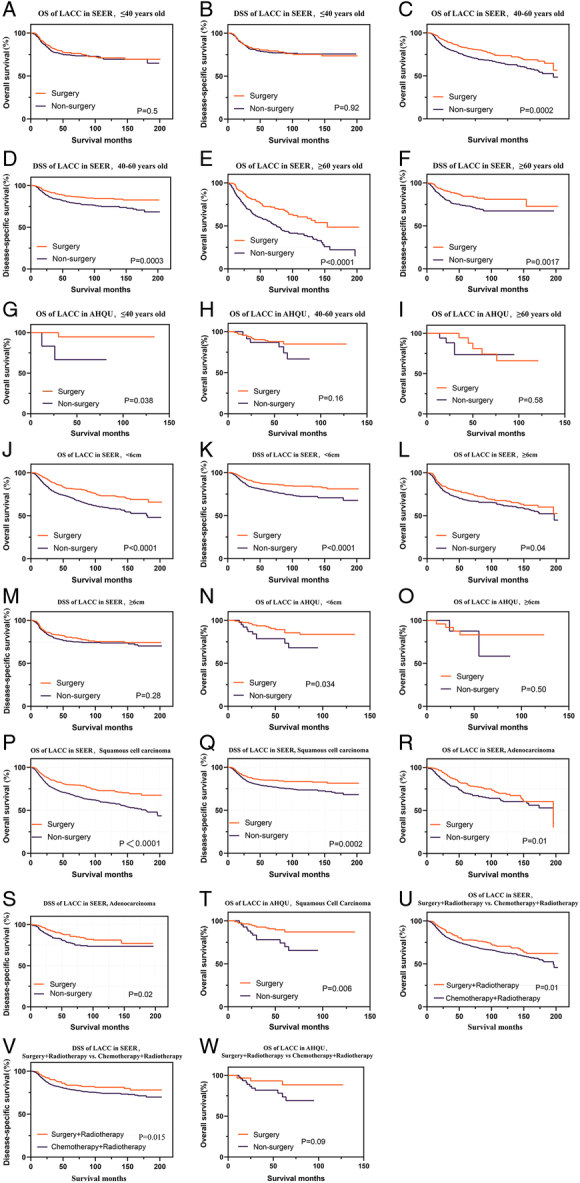
Survival curves for patients with LACC in different stratified analyses. (A, B) OS and DSS of patients with LACC aged ≤40 years in the SEER database; (C, D) OS and DSS of patients with LACC aged 40–60 years in the SEER database; (E, F) OS and DSS of patients with LACC aged ≥60 years in the SEER database; (G) OS of patients with LACC aged ≤40 years in the Affiliated Hospital of Qingdao University (AHQU) database; (H) OS of patients with LACC aged 40–60 years in AHQU database; (I) OS of patients with LACC aged ≥60 years in the AHQU database; (J, K) OS and DSS of patients with LACC with tumor diameter <6 cm in the SEER database; (L, M) OS and DSS of patients with LACC with tumor diameter ≥6 cm in the SEER database; (N) OS of patients with LACC with tumor diameter <6 cm in the AHQU database; (O) OS of patients with LACC with tumor diameter ≥6 cm in the AHQU database; (P, Q) OS and DSS of patients with LACC with cervical squamous cell carcinoma in the SEER database; (R, S) OS and DSS of patients with LACC with cervical adenocarcinoma in the SEER database; (T) OS of patients with LACC with cervical squamous cell carcinoma in the AHQU database; (U, V) OS and DSS of patients with LACC with surgery + radiotherapy vs. chemotherapy + radiotherapy in the SEER database; (W) OS of patients with LACC with surgery + radiotherapy vs. chemotherapy + radiotherapy in the AHQU database.

Multivariable Cox regression analysis identified several independent prognostic factors for OS, including age, tumor diameter, histological type, chemotherapy, FIGO stage, and surgical treatment. For DSS, independent prognostic factors included tumor diameter, histological type, chemotherapy, and surgical treatment. Compared with non-surgical treatment, surgical intervention was associated with improved OS (HR: 0.53, *P*<0.0001) and DSS (HR: 0.56, *P*<0.0001) (Table 1). Further stratified analyses indicated that surgical treatment was beneficial across different age groups, tumor diameters, and histological type (Fig. 2).

**Table 1:** Multivariable Cox analysis of the overall survival and disease-specific survival of LACC patients.

	Overall survival in SEER database		Disease-specific survival in SEER database		Overall survival in AHQU	
Variable	HR (95% CI)	*P*	HR (95% CI)	*P*	HR (95% CI)	*P*
Age						
≤40	Reference		Reference		Reference	
40–60	1.02 (0.85–1.23)	0.838	0.82 (0.66–1.01)	0.056	1.44 (0.41–5.03)	0.565
≥60	1.87 (1.54–2.28)	<0.0001	1.12 (0.89–1.43)	0.338	1.89 (0.49–7.30)	0.357
Tumor diameter						
<6 cm	Reference		Reference		Reference	
≥6 cm	1.25 (1.07–1.45)	0.004	1.30 (1.08–1.57)	0.005	1.23 (0.49–3.09)	0.657
Unknown	1.32 (0.98–1.77)	0.069	1.15 (0.77–1.70)	0.496	–	–
Rural/urban status						
Urban	Reference		Reference		Reference	
Rural	1.21 (0.93–1.56)	0.154	1.15 (0.82–1.60)	0.422	0.98 (0.48–2.03)	0.961
Income						
<$55 000	Reference		Reference		–	
$55 000–$75 000	1.36 (1.08–1.73)	0.010	1.62 (1.20–2.21)	0.002	–	–
>$75 000	1.30 (1.00–1.68)	0.047	1.64 (1.18–2.28)	0.003	–	–
Marital						
Married	Reference		Reference		Reference	
Unmarried	1.27 (1.08–1.48)	0.003	1.18 (0.98–1.42)	0.083	0.92 (0.11–7.59)	0.937
Unknown	1.06 (0.75–1.51)	0.729	0.87 (0.54–1.39)	0.548	–	–
Race						
White	Reference		Reference		–	
Black	1.20 (0.99–1.45)	0.062	1.12 (0.88–1.43)	0.366	–	–
Others	0.82 (0.64–1.06)	0.125	0.90 (0.67–1.21)	0.479	–	–
Histology						
Squamous	Reference		Reference		Reference	
Adenocarcinoma	1.05 (0.88–1.25)	0.616	1.03 (0.83–1.28)	0.809	1.56 (0.50–4.85)	0.446
Other	1.38 (1.09–1.75)	0.008	1.50 (1.14–1.99)	0.005	2.50 (0.56–11.72)	0.244
Grade						
G1–G2	Reference		Reference		Reference	
G3–G4	1.14 (0.96–1.35)	0.144	1.20 (0.97–1.48)	0.097	0.80 (0.38–1.72)	0.573
Unknown	0.91 (0.75–1.10)	0.316	0.81 (0.64–1.04)	0.095	–	–
FIGO stage						
IB3	Reference		Reference		Reference	
IIA2	1.21 (1.02–1.45)	0.033	1.19 (0.96–1.48)	0.121	1.25 (0.59–2.65)	0.567
Chemotherapy						
Yes	Reference		Reference		Reference	
No/unknown	1.63 (1.37–1.95)	<0.0001	1.25 (0.99–1.58)	0.062	0.61 (0.17–2.18)	0.442
Radiation methods						
Beam radiation	Reference		Reference		Reference	
Combination of beam radiation with implants or isotopes	0.74 (0.64–0.87)	<0.0001	0.71 (0.9–0.86)	0.001	0.64 (0.30–1.39)	0.260
Radioactive implants	0.84 (0.65–1.08)	0.166	0.70 (0.50–0.97)	0.034	–	–
Unknown	1.05 (0.56–1.99)	0.879	1.15 (0.51–2.59)	0.744	–	–
Treatment						
Non-surgery	Reference		Reference		Reference	
Surgery	0.53 (0.45–0.63)	<0.0001	0.56 (0.45–0.69)	<0.0001	0.46 (0.21–1.05)	0.064

AHQU, Affiliated Hospital of Qingdao University; CI, confidence intervals; FIGO, International Federation of Gynecology and Obstetrics; G1, well differentiated; G2, moderately differentiated; G3, poorly differentiated; G4, undifferentiated and anaplastic; HR, hazard ratios; SEER, Surveillance, Epidemiology, and End Results database.

## Discussion

Although concurrent chemoradiotherapy is currently the preferred treatment for LACC, the failure rate of this approach can reach up to 30%^6^. While NCCN and FIGO guidelines prioritize chemoradiotherapy, they do not exclude the potential role of radical surgery in the treatment of LACC. The findings of this study suggest that surgery combined with radiotherapy may provide a significant advantage in improving OS and DSS for LACC patients, particularly for those with tumors less than 6 cm in diameter. Moreover, surgical intervention demonstrated substantial survival benefits for patients across various ages and stages of disease. In conclusion, for patients with locally advanced cervical cancer, surgery combined with radiotherapy may represent a superior treatment option, especially for those with smaller tumors.

## Ethical approval

All the data used in this study were obtained from the publicly accessible SEER database and, therefore, did not require approval from a local ethics committee. Our clinical data conformed to the provisions of the Declaration of Helsinki as revised in 2013. Our data analysis was approved by the Institutional Review Board of the Affiliated Hospital of Qingdao University (IRB no: QYFY WZLL 28858). Written informed consent was obtained from all the participants.

## Consent

Not applicable.

## Source of funding

This study was supported by the Qingdao Municipal Science and Technology Bureau (Grant No. 22-3-7-smjk-15-nsh) and the Natural Science Foundation of Shandong Province (Grant No. ZR2019MH121).

## Author contribution

X.G.: formal analysis and wrote the original manuscript; Y.K.: analyzed the data and revised the manuscript; Y.N.: collected and analyzed the data; T.T. and K.L.: provided design improvement; Z.C.: resources, project administration, provided design improvement, administrative and material support, and supervised the study.

## Conflicts of interest disclosure

The authors declare no conflicts of interest.

## Research registration unique identifying number (UIN)

1. Name of the registry: Research Registry.
2. Unique identifying number or registration ID: researchregistry10318.
3. Hyperlink to your specific registration (must be publicly accessible and will be checked): https://www.researchregistry.com/browse-theregistry#home/registrationdetails/664c0ec4aa72c50028044999/

## Guarantor

Zhumei Cui had full access to all of the data in the study and take responsibility for the integrity of the data and accuracy of the data analysis.

## Data availability statement

All data used in this study can be freely accessed from the SEER program (https://seer.cancer.gov/). All raw data were collected from the open SEER database. Research data for this study are available upon request by contacting the authors.

## Provenance and peer review

Not commissioned, externally peer-reviewed.
